# West Va. State Dental Society

**Published:** 1892-06

**Authors:** 


### CORRESPONDENCE.

Morgantown, W. Va.

Editor Dental Register:

*Dear Sir*:—Please give space in your valuable journal for the following, viz.: The West Virginia State Dental Society was organized at Wheeling, January 7. Constitution and By-laws were adopted and Code of Ethics of the American Dental Association. The next annual meeting of the society will be held in Wheeling on the first Wednesday in October, 1892. H. H. Harrison, President, Wheeling, W. Va.; Geo. I. Keener, Secretary, Morgantown, W. Va.

